# Preferences and willingness for starting daily, on-demand, and long-acting injectable HIV pre-exposure prophylaxis among transfeminine persons in the US, 2022–2023

**DOI:** 10.1371/journal.pone.0320961

**Published:** 2025-04-01

**Authors:** Duygu Islek, Travis Sanchez, Stefan Baral, Carolyn Brown, Joanna A. Caldwell, Jennifer L. Glick, Danielle Friedman Nestadt, Jeb Jones, Iaah L. Lucas, Supriya Sarkar, Annemiek de Ruiter, Patrick S Sullivan, Mariah Valentine-Graves, Savannah Winter, Vani Vannappagari

**Affiliations:** 1 Rollins School of Public Health, Emory University, Atlanta, Georgia, United States of America; 2 Johns Hopkins School of Public Health, Johns Hopkins University, Baltimore, Maryland, United States of America; 3 ViiV Healthcare, Durham, North Carolina, United States of America; 4 Community Health Science & Policy, Louisiana State University Health Sciences Center, New Orleans, Louisiana, United States of America; 5 ViiV Healthcare, London, United Kingdom; RAND Corporation, UNITED STATES OF AMERICA

## Abstract

**Background:**

There remains limited information concerning US transfeminine persons preferences across varying PrEP options. We examined PrEP option willingness, preferences, and associated factors among a US nationwide sample of transfeminine persons.

**Methods:**

Sexually active transfeminine persons age 15 + were recruited online between June 2022-October 2023 through social media advertisements. Transfeminine persons not diagnosed with HIV were asked about willingness to initiate, and ranked preference, of three PrEP options– daily oral (DO), on-demand, and long-acting injectable (LA-PrEP). Log-binomial models were used to examine PrEP modality willingness and associated sociodemographic and behavioral characteristics.

**Results:**

Among 2657 transfeminine persons not currently using PrEP, 51% reported willingness to start at least one PrEP option. The highest willingness was reported for on-demand PrEP (42.6%), followed by DO (38.1%) and LA PrEP (27.1%). LA PrEP was ranked the most preferred PrEP option among transfeminine persons who reported willingness to start multiple PrEP options (43%, 277/651). Willingness to start on-demand PrEP was higher among participants who were 15-24 years, resided in the South, did not have health insurance, had more than one sex partners and reported prior awareness of on-demand PrEP. Willingness to use DO and LA PrEP was higher among those who had both private and public insurance, reported condomless anal sex, had more than one sex partners and had used non-injection illicit drugs. Additional factors for DO and LA PrEP willingness was residing in the South and prior awareness of LA PrEP, respectively.

**Conclusions:**

Among transfeminine persons, the highest willingness was reported for on-demand PrEP; and LA PrEP was ranked the most preferred option among those who reported willingness to try multiple options. Offering a variety of PrEP options, informed by an understanding of individual preferences and socio-demographic and behavioral differences, can increase overall PrEP uptake and help meet diverse needs of the transfeminine community.

## Introduction

Transfeminine persons are disproportionately impacted by HIV[1] and have been designated as one of the five priority populations in the United States under the National HIV/AIDS Strategy[2]. Daily oral (DO) HIV pre-exposure prophylaxis (PrEP) was proven to be an effective strategy to prevent HIV acquisition among transfeminine persons nearly a decade ago[3]. However, PrEP usage prevalence among transfeminine persons is estimated to be very low among transfeminine people in the US, ranging between 9% to 32% based on local estimates[4,5], and no national estimates of prevalence are available. Furthermore, among transfeminine persons who start PrEP, adherence and persistence are both relatively low, compromising PrEP’s protective efficacy[6–8].

Alternative PrEP formulations, such as long-acting injectable PrEP (LA PrEP) and oral on-demand PrEP, may enhance PrEP uptake among transfeminine persons. LA PrEP was approved by the U.S. Food and Drug Administration (FDA) in 2021 and has demonstrated superior effectiveness in preventing HIV acquisition by transfeminine individuals compared to DO PrEP [9,10]. On-demand PrEP is not FDA-approved or recommended by the CDC but has also been shown to reduce HIV transmission risk[11,12]. On-demand and LA PrEP may present opportunities to improve initiation and adherence rates among transfeminine persons. Particularly, the administration of LA PrEP via bimonthly injections may address adherence issues among transfeminine individuals potentially enhancing its overall effectiveness[8]. Furthermore, LA PrEP could offer increased privacy compared to daily oral PrEP, and possibly help reduce PrEP- related stigma for transfeminine persons [13]. On-demand PrEP might improve uptake and adherence, particularly among transfeminine individuals who are apprehensive about side effects, find it challenging to maintain a consistent daily pill routine, or desire a reduced pill burden[14]. However, there is scarce information regarding the preferences of transfeminine persons for different PrEP options. Gaining insights into these preferences can inform clinical discussions between healthcare providers and patients and may help increase overall PrEP uptake among transfeminine persons.

Certain socio-behavioral factors might also influence the preference for specific PrEP regimens among transfeminine individuals. Previous research has highlighted associations between PrEP use and socio-behavioral factors like age, race, geographic location, the number of sexual partners, engagement in condomless sex, history of sexually transmitted infections, and illicit drug use[5,15,16]. Nonetheless, the impact of these socio-demographic and behavioral factors on PrEP option preferences remains underexplored. In this study, we aimed to investigate the relative preferences for starting different PrEP options and associations between option-specific PrEP willingness and socio-demographic and behavioral characteristics among a nationwide online sample of transfeminine individuals in the United States.

## Methods

### Study design and analytic sample

The Transgender Women’s Internet Survey and Testing (TWIST) is a cross-sectional online survey of transfeminine persons in the United States[17–19]. This survey collects information on demographics, sexual behaviors, substance use, HIV and STI testing and diagnosis, and the utilization of HIV prevention services. The TWIST survey has been conducted annually since 2022. Participants were recruited through banner advertisements displayed on websites, social networking sites and smartphone applications used by transfeminine persons. Participants who were screened for the online American Men’s Internet Survey (AMIS)[20] but did not meet the eligibility criteria due to identifying as transgender women or transfeminine nonbinary were also contacted if they provided an email address and consent for future communication. Participants who clicked on an advertisement or used the provided email survey link were directed to an eligibility screener that contained questions pertaining to the inclusion criteria. Individuals who were at least 15 years of age; assigned male sex at birth; identify as female, transgender women, and/or transfeminine nonbinary people, ever had oral, anal or vaginal sex and currently reside in the United States were eligible to participate in the study. Those who meet the eligibility requirements were then taken to the online informed consent page. For the current analysis, we used data collected between June 2022 and October 2023. To form the analytic sample, we applied additional eligibility criteria, including having oral, anal, or vaginal sex in the past 12 months and no self-reported prior HIV diagnosis.

### Outcome measures

First, participants were provided with a concise overview of the DO, on-demand and LA PrEP options (S1 Table). Participants who were not currently taking PrEP were asked about starting PrEP: *“If [PrEP option] were available from your local doctor and you could access it for free, would you go to your doctor in the next month to start [PrEP option]?”* Participants who were currently using DO PrEP were asked about switching to on-demand or LA PrEP: *“If [PrEP option] were available from your local doctor and you could access it for free, would you go to your doctor in the next month to start [PrEP option]?”* If participants expressed a willingness to start multiple PrEP options, they were then asked to rank their selected PrEP options in order of preference.

### Covariate measures

Participants were asked about their sociodemographic characteristics including age (categorized as 15-24, 25-29, 30-30, 40+ years), race/ethnicity (grouped as Non-Hispanic Black, Hispanic/Latino, Non-Hispanic White, other/multiple racial groups), health insurance type (categorized as private only, public only, both private and public, other, no insurance), urbanicity of county of residence (based on self-reported residential ZIP code and categorized as large central metro, large fringe metro, medium metro, small metro and micropolitan)[21], and census region (Northeast, Midwest, South, West). Private insurance was used as the reference group in the statistical models since, under the Affordable Care Act[22], most private health insurance plans are required cover PrEP as a preventive service without any out-of-pocket expenses. In contrast, cost-sharing requirements for public insurance can differ depending on the state and therefore, individuals with private insurance may have easier access to PrEP compared to those who are uninsured or have public insurance.

Participants were asked about their behavioral characteristics in the past 12 months with items for condomless anal sex (yes/no), condomless vaginal sex (yes/no), number of sex partners (one/two or more), self-reported sexually transmitted infection (STI) diagnosis (yes/no), marijuana use (yes/no), and any illicit non-injection drug use other than marijuana(yes/no).

Prescribed medication history of participants was determined with items asking whether they are currently taking any daily prescription pills (yes/no) and whether they had any injection of prescribed medication other than a vaccine in past 12 months and, if so, who administered the prescribed injection. (Grouped as: No, I did not get any injection/Yes, injected myself/Yes, someone else gave the injection/Yes, injected myself and by someone else).

Participants were asked about their prior awareness of a particular PrEP option with the item: “*Before today, have you ever heard of [PrEP option]?*”. PrEP-use characteristics among the current DO PrEP users were described with items on current DO PrEP prescription medication brand (Truvada, Descovy ^©^), how many doses of DO PrEP they took in last 30 days (grouped as ≤ 15 doses, 16-29 doses, 30 doses) and how many months in a row they had been taking DO PrEP (grouped as <  2 months, 2-6 months, 7-12 months, 12 months and more).

### Statistical analysis

We tabulated the sociodemographic, behavioral characteristics and prescribed medication history in the analytic sample and examined the distribution of willingness to start each PrEP option by participant characteristics among those who did not use PrEP in the past 12 months and among those who were currently using DO PrEP. We described the distribution of the PrEP option ranking among participants who were willing to start multiple PrEP options.

Log-binomial regression models were used to examine the associations of all covariates with the willingness to start each PrEP option; associations are reported as unadjusted and adjusted prevalence ratios and 95% confidence intervals (CI). A similar analytic modelling approach was not possible for participants who were currently using PrEP due to small cell sizes. Therefore, we only report these data descriptively. We conducted the data analysis with SAS 9.4.

### Ethics statement

The study was conducted in compliance with federal regulations governing protection of human subjects and was reviewed and approved by Emory University’s institutional review board (Emory IRB Number: IRB00108784). Eligible participants were shown a consent form to review and asked whether they wish to participate in the survey. Participants indicated their consent to participate by clicking the statement “I consent to participate in the survey”. Participants had the option of saving a printable PDF version of the consent documents via link at the bottom of the online consent screen. Informed consent was obtained from all individual participants in the study. Participants who consented to participate in the study were taken immediately to the first screen of the survey. Participants who consented and completed the online survey were provided with a $10 e-gift card. Those who did not consent were taken to a screen thanking them for their interest and no further information was collected. Parental consent for this project was waived for inclusion of persons 15-17 years of age by the ethics committee. The study data are non-identifiable and don’t pose a risk to loss of confidentiality.

## Results

Of the 3007 participants in the analytic sample, 87% were < 40 years old, 72% were Non-Hispanic White, 10% were Hispanic/Latino and 7% were Non-Hispanic Black persons. (S2 Table). Most participants had private health insurance, had some college/technical postgraduate degree and over half were employed in full-time jobs. A third of participants resided in the South and 40% of participants resided in large central metro areas. Approximately 42% of participants had condomless anal or vaginal sex and about half of participants had more than one sex partner in the past 12 months. Seventy one percent of the participants were taking daily prescription pills. Also, 37% reported having a prescribed injection medication in past 12 months. Twenty four percent of the participants had injected prescribed medication by themselves. Overall, 12% (n = 350) had used DO PrEP in the past 12 months and 11% (n = 275) were current DO PrEP users. Among participants who did not use PrEP in the past 12 months (n = 2657), 32% (n = 841) reported ever using DO PrEP.

**Fig 1:**
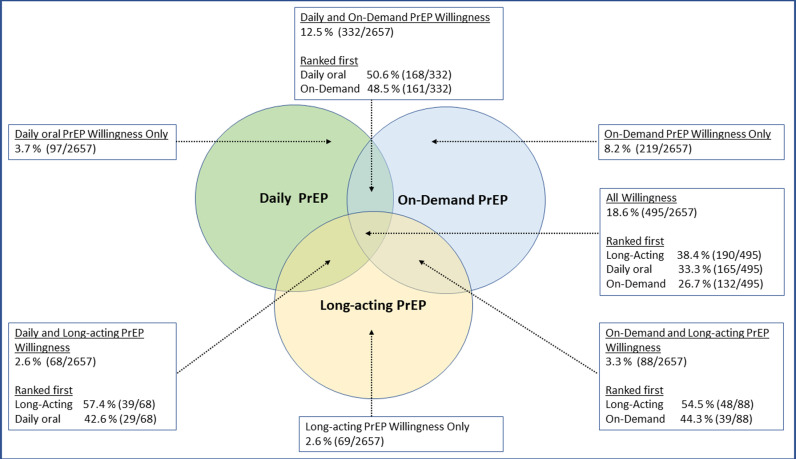
Willingness and relative preferences to start PrEP options among transfeminine persons who did not use PrEP in the past 12 months, Transgender Women’s Internet Survey and Testing (TWIST), 2022-23.

Among 2657 participants who did not use PrEP in the past 12 months, 51% (n = 1368) were willing to start at least one PrEP option. The highest level of willingness to start was for on-demand PrEP, with 43% of participants expressing interest in this option. This was followed by DO PrEP (38.1%) and LA PrEP (27%). Overall, 19% of participants were willing to start multiple PrEP options and among those participants, LA PrEP was ranked the most preferred PrEP option (Fig 1**).** Among participants who ever used PrEP and did not use PrEP in the past 12 months (n = 841), 39% expressed willingness to use on-demand PrEP, followed by 36% to use DO and 25% to use LA PrEP.

In multivariable modeling of participants who had not used PrEP in the past 12 months, willingness to start on-demand PrEP was higher in multivariable modeling among participants who were 15-24 years, resided in the South, had no health insurance, had more than one sex partner, and had heard of on-demand PrEP. Participants were less likely to be willing to start on-demand PrEP if they were using daily prescription pills and if they had condomless vaginal sex (Table 1**).**

**Table 1:** Willingness to use on-demand PrEP among transfeminine persons who did not use PrEP in the past 12 months, TWIST, 2022-23.

	Willing to use on-demand PrEP n (%)	Not willing to use on-demand PrEP or not sure n (%)	Unadjusted prevalence ratio and 95% confidence interval	Adjusted prevalence ratio and 95% confidence interval
**Total**	1134 (44.5)	1412 (55.5)		
**Age (years)**				
15-24	500 (49.5)	510 (50.5)	**1.40 (1.20, 1.65)**	**1.20 (1.02, 1.41)**
25-29	231 (42.3)	315 (57.7)	**1.20 (1.01, 1.43)**	1.05 (0.88, 1.25)
30-39	288 (43.4)	376 (56.6)	**1.23 (1.04, 1.46)**	1.10 (0.93, 1.30)
40 ^+^	115 (35.3)	211 (64.7)	ref	ref
**Race/Ethnicity**				
Black, non-Hispanic	55 (34.8)	103 (65.2)	0.77 (0.62, 0.96)	0.78 (0.63, 0.97)
Hispanic or Latino	126 (48.5)	134 (51.5)	1.07 (0.94, 1.23)	0.99 (0.87, 1.14)
White, non-Hispanic	835 (45.2)	1012 (54.8)	ref	ref
Other or multipl races	105 (40.2)	156 (59.8)	0.89 (0.76, 1.04)	0.85 (0.73, 0.99)
**Health insurance**				
None	112 (53.1)	99 (46.9)	**1.22 (1.06, 1.40)**	**1.17 (1.03, 1.34)**
Private only	701 (43.5)	909 (56.5)	ref	ref
Public only	204 (40.6)	298 (59.4)	0.93 (0.83, 1.05)	0.95 (0.84, 1.07)
Both public and private	73 (57.0)	55 (43.0)	**1.31 (1.12, 1.54)**	1.17 (1.00, 1.36)
Other	32 (49.2)	33 (50.8)	1.13 (0.88, 1.46)	1.06 (0.84, 1.36)
**NCHS rural-urban category**				
Large central metro	436 (44.6)	542 (55.4)	ref	ref
Large fringe metro	226 (42.6)	305 (57.4)	0.95 (0.85, 1.08)	0.96 (0.85, 1.08)
Medium metro	255 (46.4)	294 (53.6)	1.04 (0.93, 1.17)	1.01 (0.91, 1.12)
Small metro, micropolitan and non-core	215 (44.4)	269 (55.6)	1.00 (0.88, 1.13)	1.04 (0.92, 1.17)
**Census region**				
Northeast	179 (42.3)	244 (57.7)	ref	ref
Midwest	235 (41.7)	328 (58.3)	0.99 (0.85, 1.14)	1.03 (0.89, 1.19)
South	398 (47.8)	434 (52.2)	1.13 (0.99, 1.29)	**1.17 (1.03, 1.33)**
West	321 (44.2)	406 (55.8)	1.04 (0.91, 1.20)	1.03 (0.90, 1.18)
**STI diagnosis in past 12 months**				
No	1085 (44.0)	1380 (56.0)	ref	ref
Yes	49 (60.5)	32 (39.5)	**1.37 (1.15, 1.65)**	1.16 (0.95, 1.42)
**Condomless anal sex in past 12 months**				
No	655 (43.1)	863 (56.9)	ref	ref
Yes	479 (46.6)	549 (53.4)	1.08 (0.99, 1.18)	1.05 (0.96, 1.15)
**Condomless vaginal sex in past 12 months**				
No	684 (47.4)	759 (52.6)	ref	ref
Yes	450 (40.8)	653 (59.2)	**0.86 (0.79, 0.94)**	**0.88 (0.80, 0.96)**
**Number of partners**				
One	461 (32.9)	942 (67.1)	ref	ref
More than one	659 (59.6)	447 (40.4)	**1.81 (1.66, 1.98)**	**1.68 (1.53, 1.85)**
**Marijuana use in past 12 months**				
No	644 (42.5)	870 (57.5)	ref	ref
Yes	490 (47.5)	542 (52.5)	**1.12 (1.02, 1.22)**	1.00 (0.90, 1.10)
**Other non-injection illicit drug use in past 12 months**				
No	795 (41.9)	1102 (58.1)	ref	ref
Yes	339 (52.2)	310 (47.8)	**1.25 (1.14, 1.36)**	1.06 (0.96, 1.18)
**Taking daily prescription pills**				
No	351 (46.5)	404 (53.5)	ref	ref
Yes	773 (43.6)	1001 (56.4)	0.94 (0.85, 1.03)	0.94 (0.85, 1.03)
**Injection of prescribed medication in past 12 months**				
No	712 (42.2)	975 (57.8)	ref	ref
Yes, I injected myself	260 (46.6)	298 (53.4)	1.10 (0.99, 1.23)	1.07 (0.97, 1.18)
Yes, someone else gave me the injection	112 (52.6)	101 (47.4)	**1.25 (1.08, 1.43)**	1.04 (0.91, 1.19)
Yes, injected myself and by someone else	38 (52.1)	35 (47.9)	1.23 (0.98, 1.55)	1.02 (0.82, 1.27)
**Heard of on-demand PrEP**				
No	852 (42.5)	1151 (57.5)	ref	ref
Yes	279 (51.9)	259 (48.1)	**1.22 (1.11, 1.34)**	**1.13 (1.04, 1.24)**

Abbreviations: NCHS: National Center for Health Statistics, PrEP: Pre-exposure prophylaxis.

* Age, race/ethnicity, health insurance, NCHS rural-urban category, census region, STI diagnosis in past 12 months, condomless anal sex in past 12 months, condomless vaginal sex in past 12 months, number sex partners, marijuana use in past 12 months, non-injection illicit drug use past 12 months, taking daily prescription pills, injection of prescribed medication in past 12 months, prior awareness of on demand PrEP were included in the models to estimate adjusted prevalence ratios.

** Bold text indicates statistical significance.

*** Data does not add up to the total number of participants due to missing information resulting from non-response from some of the participants

Willingness to use DO PrEP was higher among participants who had both private and public insurance, resided in the South, reported condomless anal sex and more than one sex partner, and had used non-injection illicit drugs. Participants who were taking daily prescription pills and who had condomless vaginal sex were less likely to be willing to start DO PrEP, although this association was not significant in multivariable models (Table 2).

**Table 2:** Willingness to use daily oral PrEP among United States transfeminine persons who did not use PrEP in the past 12 months, TWIST, 2022-23.

	Willing to use daily oral PrEP n (%)	Not willing to use daily oral PrEP or not sure n (%)	Unadjusted prevalence ratio and 95% confidence interval	Adjusted prevalence ratio and 95% confidence interval
**Total**	992 (39%)	1549 (61%)		
**Age (years)**				
15-24	426 (42.3)	582 (57.7)	**1.26 (1.07, 1.50)**	1.08 (0.91, 1.28)
25-29	203 (37.1)	344 (62.9)	1.11 (0.92, 1.34)	1.00 (0.83, 1.21)
30-39	254 (38.5)	406 (61.5)	1.15 (0.96, 1.38)	1.06 (0.88,1.27)
40^ + ^	109 (33.4)	217 (66.6)	ref	ref
**Race/Ethnicity**				
Black, non-Hispanic	52 (33.1)	105 (66.9)	0.84 (0.67, 1.06)	0.78 (0.61, 0.98)
Hispanic or Latino	109 (42.1)	150 (57.9)	1.07 (0.92, 1.25)	0.97 (0.83, 1.14)
White, non-Hispanic	726 (39.3)	1119 (60.7)	ref	ref
Other or multiple races	94 (36.0)	167 (64.0)	0.92 (0.77, 1.09)	0.87 (0.73, 1.02)
**Health insurance**				
None	98 (46.9)	111 (53.1)	**1.29 (1.10, 1.51)**	1.16 (0.99, 1.36)
Private only	585 (36.4)	1022 (63.6)	ref	ref
Public only	202 (40.2)	301 (59.8)	1.10 (0.97, 1.25)	1.08 (0.96, 1.23)
Both private and public	69 (53.5)	60 (46.5)	**1.47 (1.24, 1.75)**	**1.29 (1.09, 1.52)**
Other	24 (38.1)	39 (61.9)	1.05 (0.76, 1.44)	1.01 (0.74, 1.37)
**NCHS rural-urban category**				
Large central metro	373 (38.3)	602 (61.7)	ref	ref
Large fringe metro	189 (35.6)	342 (64.4)	0.93 (0.81, 1.07)	0.93 (0.81, 1.07)
Medium metro	223 (40.8)	324 (59.2)	1.07 (0.94, 1.21)	1.05 (0.92, 1.19)
Small metro, micropolitan	205 (42.4)	279 (57.6)	1.11 (0.97, 1.26)	1.09 (0.95, 1.24)
**Census region**				
Northeast	146 (34.6)	276 (65.4)	ref	ref
Midwest	216 (38.6)	344 (61.4)	1.11 (0.94, 1.32)	1.11 (0.94, 1.32)
South	356 (42.9)	474 (57.1)	**1.24 (1.06, 1.44)**	**1.25 (1.08, 1.46)**
West	273 (37.5)	455 (62.5)	1.08 (0.92, 1.27)	1.07 (0.91, 1.25)
**STI diagnosis in in past 12 months**				
No	945 (38.4)	1513 (61.6)	ref	ref
Yes	47 (56.6)	36 (43.4)	**1.47 (1.21, 1.79)**	1.18 (0.95, 1.47)
**Condomless anal sex in past 12 months**				
No	550 (36.3)	967 (63.7)	ref	ref
Yes	442 (43.2)	582 (56.8)	**1.19 (1.08, 1.31)**	**1.13 (1.02, 1.24**)
**Condomless vaginal sex in past 12 months**				
No	602 (41.7)	840 (58.3)	ref	ref
Yes	390 (35.5)	709 (64.5)	**0.85 (0.77, 0.94)**	**0.89 (0.81, 0.99)**
**Number of partners**				
One	399 (28.5)	1001 (71.5)	ref	ref
More than one	577 (52.3)	527 (47.7)	**1.83 (1.66, 2.03)**	**1.74 (1.56, 1.94)**
**Marijuana use in past 12 months**				
No	570 (37.7)	942 (62.3)	ref	ref
Yes	422 (41.0)	607 (59.0)	1.09 (0.99, 1.20)	0.98 (0.87, 1.10)
**Other non-injection illicit drug use in past 12 months**				
No	692 (36.6)	1201 (63.4)	ref	ref
Yes	300 (46.3)	348 (53.7)	**1.27 (1.14, 1.40**)	**1.08 (0.96, 1.22)**
**Taking daily prescription pills**				
No	326 (43.2)	428 (56.8)	ref	ref
Yes	657 (37.1)	1113 (62.9)	**0.86 (0.78, 0.95)**	0.90 (0.81, 1.01)
**Injection of prescribed medication in past 12 months**				
No	638 (37.9)	1044 (62.1)	ref	ref
Yes, I injected myself	217 (38.9)	341 (61.1)	1.03 (0.91, 1.16)	0.99 (0.88, 1.11)
Yes, someone else gave me the injection	98 (46.0)	115 (54.0)	**1.21 (1.04, 1.42)**	1.02 (0.87, 1.18)
Yes, injected myself and by someone else	30 (41.7)	42 (58.3)	1.10 (0.83, 1.45)	0.90 (0.68, 1.18)
**Heard of daily oral PrEP**				
No	301 (40.5)	442 (59.5)	ref	ref
Yes	691 (38.4)	1107 (61.6)	0.95 (0.85, 1.05)	0.98 (0.88, 1.09)

Abbreviations: NCHS: National Center for Health Statistics, PrEP: Pre-exposure prophylaxis.

* Age, race/ethnicity, health insurance, NCHS rural-urban category, census region, STI diagnosis in past 12 months, condomless anal sex in past 12 months, condomless vaginal sex in past 12 months, number sex partners, marijuana use in past 12 months, non-injection illicit drug use past 12 months, taking daily prescription pills, injection of prescribed medication in past 12 months, prior awareness of daily oral PrEP were included in the models to estimate adjusted prevalence ratios.

** Bold text indicates statistical significance.

*** Data does not add up to the total number of participants due to missing information resulting from non-response from some of the participants

Willingness to start LA PrEP was higher in multivariable modeling among participants who had both private and public insurance, resided in the South, had condomless anal sex, had more than one sex partner, had used non-injection illicit drugs and had heard of LA PrEP (Table 3**).**

**Table 3:** Willingness to use long-acting injectable PrEP among transfeminine persons who did not use PrEP in the past 12 months, TWIST, 2022-23.

	Willing to use LAI PrEP n (%)	Not willing to use LAI PrEP or not sure n (%)	Unadjusted prevalence ratio and 95% confidence interval	Adjusted prevalence ratio and 95% confidence interval
**Total**	720 (28.3)	1825 (71.7)		
**Age (years)**				
15-24	291 (28.8)	720 (71.2)	1.05 (0.86, 1.29)	0.95 (0.78, 1.17)
25-29	146 (26.7)	400 (73.3)	0.98 (0.78, 1.23)	0.83 (0.66, 1.04)
30-39	194 (29.3)	468 (70.7)	1.07 (0.87, 1.33)	0.92 (0.75, 1.14)
40^ + ^	89 (27.3)	237 (72.7)	ref	ref
**Race/Ethnicity**				
Black, non-Hispanic	44 (27.8)	114 (72.2)	0.97 (0.75, 1.26)	0.92 (0.71, 1.20)
Hispanic or Latino	77 (29.6)	183 (70.4)	1.03 (0.84, 1.26)	0.93 (0.76, 1.15)
White, non-Hispanic	532 (28.8)	1316 (71.2)	ref	ref
Other or multipl races	62 (23.9)	197 (76.1)	0.83 (0.66, 1.05)	0.80 (0.64, 1.00)
**Health insurance**				
None	66 (31.4)	144 (68.6)	1.20 (0.97, 1.49)	1.15 (0.93, 1.43)
Private only	421 (26.2)	1188 (73.8)	ref	ref
Public only	154 (30.5)	351 (69.5)	1.17 (1.00, 1.36)	1.10 (0.94, 1.28)
Both private and public	59 (45.7)	70 (54.3)	**1.75 (1.42, 2.15)**	**1.56 (1.28, 1.91)**
Other	15 (23.8)	48 (76.2)	0.91 (0.58, 1.43)	0.96 (0.62, 1.48)
**NCHS rural-urban category**				
Large central metro	267 (27.4)	709 (72.6)	ref	ref
Large fringe metro	135 (25.4)	396 (74.6)	0.93 (0.78, 1.11)	0.94 (0.79, 1.12)
Medium metro	170 (31.1)	376 (68.9)	1.14 (0.97, 1.34)	1.08 (0.92, 1.27)
Small metro, micropolitan and non-core	148 (30.3)	340 (69.7)	1.11 (0.94, 1.31)	1.09 (0.92, 1.30)
**Census region**				
Northeast	113 (26.8)	309 (73.2)	ref	ref
Midwest	147 (26.0)	418 (74.0)	0.97 (0.79, 1.20)	0.99 (0.80, 1.22)
South	265 (31.9)	565 (68.1)	1.19 (0.99, 1.44)	1.20 (1.00, 1.44)
West	195 (26.8)	532 (73.2)	1.00 (0.82, 1.22)	0.98 (0.81, 1.20)
**STI diagnosis in past 12 months**				
No	690 (28.0)	1774 (72.0)	ref	ref
Yes	30 (37.0)	51 (63.0)	1.32 (0.99, 1.77)	1.24 (0.94, 1.63)
**Condomless anal sex in past 12 months**				
No	387 (25.5)	1130 (74.5)	ref	ref
Yes	333 (32.4)	695 (67.6)	**1.27 (1.12, 1.44)**	**1.16 (1.03, 1.32)**
**Condomless vaginal sex in past 12 months**				
No	417 (28.8)	1029 (71.2)	ref	ref
Yes	303 (27.6)	796 (72.4)	0.96 (0.84, 1.08)	0.95 (0.84, 1.08)
**Number of partners**				
One	281 (20.1)	1120 (79.9)	ref	ref
More than one	430 (38.9)	676 (61.1)	**1.94 (1.71, 2.20)**	**1.79 (1.57, 2.04)**
**Marijuana use in past 12 months**				
No	421 (27.8)	1096 (72.2)	ref	ref
Yes	299 (29.1)	729 (70.9)	1.05 (0.92, 1.19)	0.93 (0.81, 1.07)
**Other non-injection illicit drug use in past 12 months**				
No	494 (26.0)	1404 (74.0)	ref	ref
Yes	226 (34.9)	421 (65.1)	**1.34 (1.18, 1.53)**	**1.22 (1.06, 1.41)**
**Taking daily prescription pills**				
No	230 (30.5)	523 (69.5)	ref	ref
Yes	483 (27.2)	1292 (72.8)	0.89 (0.78, 1.02)	0.92 (0.80, 1.06)
**Injection of prescribed medication in past 12 months**				
No	428 (25.4)	1259 (74.6)	ref	–
Yes, I injected myself	176 (31.5)	382 (68.5)	**1.24 (1.07, 1.44)**	–
Yes, someone else gave me the injection	80 (37.7)	132 (62.3)	**1.49 (1.23, 1.80)**	–
Yes, injected myself and by someone else	29 (39.7)	44 (60.3)	**1.57 (1.17, 2.10)**	–
**Heard of LA PrEP**				
No	521 (26.1)	1479 (74.0)	ref	ref
Yes	197 (36.5)	343 (63.5)	**1.40 (1.23, 1.60)**	**1.28 (1.12, 1.47)**

Abbreviations: NCHS: National Center for Health Statistics, PrEP: Pre-exposure prophylaxis, LA PrEP: long acting injectable PrEP.

* Age, race/ethnicity, health insurance, NCHS rural-urban category, census region, STI diagnosis in past 12 months, condomless anal sex in past 12 months, condomless vaginal sex in past 12 months, number sex partners, marijuana use in past 12 months, non-injection illicit drug use past 12 months, taking daily prescription pills, injection of prescribed medication in past 12 months, prior awareness of long-acting injectable PrEP were included in the models to estimate adjusted prevalence ratios.

** Bold text indicates statistical significance.

*** Data does not add up to the total number of participants due to missing information resulting from non-response from some of the participants

**Fig 2:**
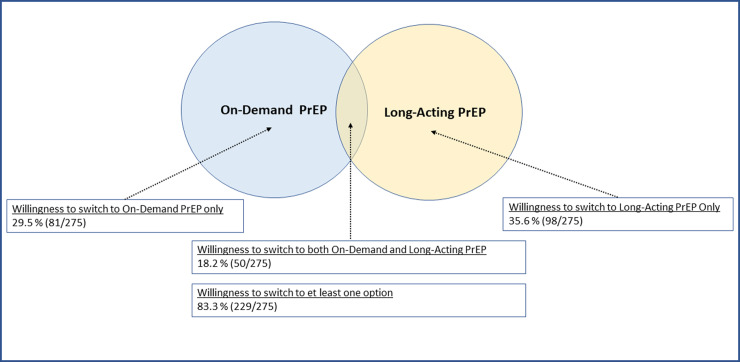
Willingness and relative preferences to switch to other PrEP options among transfeminine persons who are current daily oral PrEP users, Transgender Women’s Internet Survey and Testing (TWIST), 2022-23.

Among 275 participants who were currently using DO PrEP, only 112 (41%) were fully adherent to the required daily dose and reported taking 30 pills in the last 30 days; 83 (30%) participants reported that they used less than 15 doses and 65 participants (24%) reported that they used between 16-29 doses. Among these participants, 83.3% (n = 229) were willing to switch to at least one other PrEP option. Willingness to switch to LA PrEP (35.6%) was higher than willingness to switch to on-demand PrEP (29.5%) (Fig 2**).** The highest proportions of willingness to switch to LA PrEP were reported by participants who were non-Hispanic Black and Hispanic; who had both private and public insurance; who had (other) prescribed medication injections; and who had prior awareness of LA PrEP (S3 Table**).** The highest proportions of willingness to switch to on-demand PrEP were among participants who were non-Hispanic Black; who had both public and private insurance; who were not taking any daily prescription pills; and who took less than 15 doses of DO PrEP in the last 30 days (S4 Table**).**

## Discussion

Transfeminine persons report substantial interest in starting PrEP when it is available for free at their local doctor’s office. Further, we noted that PrEP preferences varied by socio-behavioral factors, and that substantial numbers of transfeminine persons were willing to consider switching to different PrEP regimens or routes of administration. Our data can be used to inform strategies to improve clinical PrEP services for transfeminine persons, and perhaps increase overall uptake among transfeminine persons in the US.

PrEP preferences among transfeminine persons in our study varied by demographic characteristics, particularly by age, region of residence, and health insurance. These findings align with previous studies reporting high PrEP willingness among young transgender women between 18-29 years of age[23]. Despite the high level of willingness, PrEP uptake remains suboptimal among transfeminine persons[5] underscoring the need for specific strategies to promote PrEP uptake and retention [24]. We also observed regional variations in PrEP preferences. Participants residing in the South had a higher willingness to start on-demand, DO and LA PrEP compared to participants in the Northeast. Given the high rates of HIV among transgender women in the South[1], a high willingness to use DO and LA PrEP in this region is promising for increasing overall PrEP uptake. Having both public and private insurance was associated with a greater willingness towards using DO or LA PrEP in our study. Also, willingness to use on-demand PrEP was higher among those without health insurance as compared to having only private insurance. It is important to recognize that our survey question about PrEP willingness only asked about willingness to use PrEP if the provision of PrEP was free. Therefore, we did not account for the role of health insurance in coverage for medication in assessing respondents’ willingness to start PrEP. Including health insurance in our multivariable models, however, allowed us to gain a deeper understanding of participants’ previous experiences and perceptions of health insurance which can also be a proxy for their socioeconomic status[25]. In this context, on-demand PrEP, which would likely be associated with lower costs of medications, might be more appealing to participants without health insurance, while those with multiple insurance options may be more open to exploring various PrEP options since they have more options to pay for PrEP services[26].

Behavioral risk factors were also associated with PrEP preferences in our study. Participants with more than one sex partners and who had condomless anal sex had higher willingness to start DO and LA PrEP. These results parallel recommendations for who should consider starting PrEP and were confirmed in previous studies[27, 28]. Conversely, participants who had condomless vaginal sex were less willing to use DO or on-demand PrEP than those who had oral, anal, or vaginal sex with condoms. This could be due to the infrequent nature of vaginal sex, leading to a perception of lower risk compared to anal sex[1]. It could also be attributed to the role (i.e., insertive or receptive) in the sex act[29]. Moreover, transfeminine persons who engage in condomless vaginal sex may only do so within specific types of relationships, such as having only one main partner or spouse where HIV prevention is perceived to be less important because HIV acquisition risks are perceived to be low[29,30].

We also observed that transfeminine persons who used non-injection illicit drugs other than marijuana were more willing to start DO and LA PrEP. However, there was no significant association between marijuana use and PrEP willingness. This might be associated with a higher HIV risk perception and PrEP awareness among people using injected drugs[13] and a relatively lower risk perception among those who use marijuana^21^.

Participants’ experiences with current prescription medications were associated with their preferences for PrEP in our study. Participants who had previously taken injections in the past 12 months expressed a greater interest in using LA injectable PrEP compared to those who did not have any injection prescriptions. In particular, participants who were receiving injections from others rather than injecting themselves showed a greater interest in LA PrEP, which may be due to their lower levels of anticipated healthcare stigma, which is a common issue that hinders engagement in the PrEP care continuum among transfeminine individuals[31,32]. Similarly, DO PrEP and on-demand PrEP willingness were both higher among participants who were receiving injections and who had someone else administer their injections in univariate models, potentially indicating greater engagement with healthcare or social support since the injector could be a primary care provider or a family member[33]. Those who were taking daily prescription pills were less likely to express willingness to use DO or on-demand PrEP compared to those who were not on any oral prescriptions; however, these findings were not statistically significant in multivariable models. Further research into the role of additional healthcare experiences (i.e., gender affirming care experiences, stigma in healthcare setting) is warranted.

Prior awareness of on-demand and LA PrEP was another factor associated with a higher willingness to start to these options. Improved PrEP awareness has been shown to increase willingness to use DO PrEP in previous studies[13,27]. Health education campaigns and open dialogues between healthcare providers and patients about newer PrEP options may enhance awareness and ultimately PrEP willingness and use [34,35].

One important consideration is that nearly half of the participants report no willingness to start any of the PrEP options in our hypothetical scenario where they would be offered PrEP for free by their doctor. This could be related to various factors, including a low HIV infection risk perception[36] and anticipation of experiencing healthcare stigma[32]. To increase PrEP willingness among transfeminine individuals, tailored interventions that address the unique needs of transgender women would be needed, including targeted outreach programs that advance access to PrEP-related health education.

This study has limitations. The data were collected through online recruitment using a convenience sampling approach, which limits external generalizability. Our sample may also have a higher proportion of individuals who could benefit from PrEP due to recruitment from social media websites and apps where people seek sex partners. Additionally, the survey relied on self-reported behaviors, which may be subject to misclassification[37]. Finally, the hypothetical scenario of PrEP options being available free of charge from local doctors may not reflect real-world access barriers and may be maximal estimates for willingness to start PrEP.

## Conclusion

There is substantial interest among transfeminine persons to start on-demand, DO, and LA PrEP options. Many participants expressed willingness to start multiple PrEP options; LA PrEP was the most preferred choice among those interested in all three PrEP administration modes (i.e., oral, on-demand, and LA PrEP). Our findings underscore that PrEP preferences are associated with various socio-behavioral factors and past experiences with prescribed medications. Designing outreach programs to increase education of newer options like LA PrEP may lead to higher PrEP willingness. Offering a variety of PrEP options, informed by an understanding of individual preferences and socio-demographic and behavioral differences, can increase overall PrEP uptake and help meet the diverse needs of the transfeminine community.

## Supporting information

S1 Table Background information presented about the PrEP option (DOCX)

S2 Table Characteristics of the analytic sample, TWIST 2022-23 (N = 3007) (DOCX)

S3 Table Willingness to switch to long-acting injectable PrEP among transfeminine persons who are current oral PrEP users, TWIST, 2022-23 (DOCX)

S4 Table Willingness to switch to on-demand PrEP among transfeminine persons who are current oral PrEP users, TWIST, 2022-23 (DOCX)
