# Supplementary material for: Preferences and willingness for starting daily, on-demand, and long-acting injectable HIV pre-exposure prophylaxis among transfeminine persons in the US, 2022–2023
Source: PLoS One. 2025 Apr 1;20(4):e0320961. doi: 10.1371/journal.pone.0320961 (PMC11960950; doi:10.1371/journal.pone.0320961)
Supplement: S1 Table — (DOCX) [file pone.0320961.s001.docx]

**S1 Table.** Background information presented about the PrEP option

| LA-PrEP | A long-acting form of PrEP that is given as an injection in your buttocks once every 2 months has been found to be effective in preventing HIV infection. You would have to see a doctor or healthcare provider to start injectable PrEP and go back for check-ups once every 2 months to stay on it. The possible side effects of injectable PrEP are mild-to-moderate pain at the injection site that lasts 2 to 7 days, mild rash at the injection site that clears up on its own, or a headache that lasts a couple of days after injection. Studies have found that injectable PrEP is effective at preventing HIV infection. This approach isn’t currently approved by the FDA (Food and Drug Administration). |
| --- | --- |
| Daily Oral PrEP | PrEP stands for pre-exposure prophylaxis. Daily oral PrEP is a pill that a person who is HIV negative takes every day in order to prevent getting HIV. PrEP is safe, but some people experience side effects like diarrhea, nausea, headache, fatigue, and stomach pain. These side effects usually go away over time. You have to see a doctor or other healthcare provider to start daily oral PrEP and go back for check-ups once every 3 months to stay on it. Studies have shown that daily oral PrEP provides about 99% protection against HIV infection when it is taken every day. |
| On-demand Oral PrEP | There is another way that some people take PrEP pills called on-demand oral PrEP. On-demand oral PrEP is also known as intermittent” or “event driven” PrEP. With on-demand oral PrEP you take a series of pills around the time when you have sex. This means taking two pills 2 to 24 hours before sex, one pill 24 hours after the first dose, and one pill 24 hours after the second dose. Some studies found that on-demand PrEP is effective at preventing HIV infection. This approach isn’t currently approved by the FDA (Food and Drug Administration). |
