# Supplementary material for: Preferences and willingness for starting daily, on-demand, and long-acting injectable HIV pre-exposure prophylaxis among transfeminine persons in the US, 2022–2023
Source: PLoS One. 2025 Apr 1;20(4):e0320961. doi: 10.1371/journal.pone.0320961 (PMC11960950; doi:10.1371/journal.pone.0320961)
Supplement: S2 Table — (DOCX) [file pone.0320961.s002.docx]

**S2 Table.** Characteristics of the analytic sample, TWIST 2022-23 (N= 3007)

|  | **n** | **%** |
| --- | --- | --- |
| Total | 3007 | 100 |
| **Age (years)** |  |  |
| 15-24 | 1104 | 36.71 |
| 25-29 | 691 | 22.98 |
| 30-39 | 823 | 27.37 |
| 40+ | 389 | 12.94 |
| **Race/Ethnicity** |  |  |
| Black, non-Hispanic | 218 | 7.25 |
| Hispanic or Latino | 310 | 10.31 |
| White, non-Hispanic | 2159 | 71.8 |
| Other or multiple races | 297 | 9.88 |
| **Health insurance** |  |  |
| None | 239 | 8.0 |
| Private only | 1869 | 63.0 |
| Public only | 620 | 20.9 |
| Other | 84 | 2.8 |
| Multiple (public and private) | 157 | 5.3 |
| **Education level** |  |  |
| < HS diploma | 224 | 7.45 |
| HS diploma or equivalent | 631 | 20.98 |
| Some college or technical degree | 1110 | 36.91 |
| College degree or postgraduate education | 1036 | 34.45 |
| **Employment status** |  |  |
| Employed for wages full-time | 1516 | 50.72 |
| Employed for wages part-time | 599 | 20.04 |
| Self employed | 184 | 6.16 |
| A homemaker | 48 | 1.61 |
| Retired | 28 | 0.94 |
| Not employed | 530 | 17.73 |
| Unable to work(disabled) | 84 | 2.81 |
| **Household Income** |  |  |
| $0-19999 | 431 | 15.49 |
| $20000-39999 | 649 | 23.32 |
| $40000-74999 | 743 | 26.70 |
| $75000 or more | 960 | 34.50 |
| **NCHS rural-urban category** |  |  |
| Large central metro | 1211 | 40.27 |
| Large fringe metro | 627 | 20.85 |
| Medium metro | 613 | 20.39 |
| Small metro | 291 | 9.68 |
| Micropolitan | 173 | 5.75 |
| Non-core | 85 | 2.83 |
| **Census region** |  |  |
| Northeast | 512 | 17.03 |
| Midwest | 645 | 21.46 |
| South | 987 | 32.83 |
| West | 862 | 28.68 |
| **STI diagnosis in past 12 months** |  |  |
| No | 2870 | 95.44 |
| Yes | 137 | 4.56 |
| **Condomless anal sex in past 12 months** |  |  |
| No | 1710 | 56.87 |
| Yes | 1297 | 43.13 |
| **Condomless vaginal sex in past 12 months** |  |  |
| No | 1753 | 58.30 |
| Yes | 1254 | 41.70 |
| **Number of partners in past 12 months** |  |  |
| One | 1487 | 49.45 |
| More than one | 1473 | 48.99 |
| **Marijuana use in past 12 months** |  |  |
| No | 1773 | 58.96 |
| Yes | 1234 | 41.04 |
| **Other non-injection illicit drug use in past 12 months** |  |  |
| No | 2179 | 72.46 |
| Yes | 828 | 27.54 |
| **Used PrEP in past 12 months** |  |  |
| No | 2657 | 88.36 |
| Yes | 350 | 11.64 |
| **Taking daily prescription pills** |  |  |
| No | 881 | 29.48 |
| Yes | 2107 | 70.52 |
| **Injection of prescribed medication in past 12 months** | |  |
| No | 1889 | 63.24 |
| Yes, I injected myself | 709 | 23.74 |
| Yes, someone else gave me the injection | 277 | 9.27 |
| Yes, injected myself and by someone else | 112 | 3.75 |
