# Supplementary material for: Preferences and willingness for starting daily, on-demand, and long-acting injectable HIV pre-exposure prophylaxis among transfeminine persons in the US, 2022–2023
Source: PLoS One. 2025 Apr 1;20(4):e0320961. doi: 10.1371/journal.pone.0320961 (PMC11960950; doi:10.1371/journal.pone.0320961)
Supplement: S3 Table — (DOCX) [file pone.0320961.s003.docx]

**S3 Table**. Willingness to switch to long-acting injectable PrEP among transfeminine persons who are current oral PrEP users, TWIST, 2022-23

|  | **Willing to switch to LAI PrEP**  **n (%)** | **Not willing to switch to LAI PrEP or not sure n (%)** |
| --- | --- | --- |
| **Total** | 148 (54.0) | 126 (46.0) |
| **Age (years)** |  |  |
| 15-24 | 19 (45.2) | 23 (54.8) |
| 25-29 | 44 (48.9) | 46 (51.1) |
| 30-39 | 63 (63.0) | 37 (37.0) |
| 40+ | 22 (52.4) | 20 (47.6) |
| **Race/Ethnicity** |  |  |
| Black, non-Hispanic | 32 (66.7) | 16 (33.3) |
| Hispanic or Latino | 18 (72.0) | 7 (28.0) |
| White, non-Hispanic | 90 (50.3) | 89 (49.7) |
| Other or multipl races | 7 (33.3) | 14 (66.7) |
| **Health insurance** |  |  |
| None | 8 (80.0) | 2 (20.0) |
| Private only | 74 (48.4) | 79 (51.6) |
| Public only | 39 (52.7) | 35 (47.3) |
| Other | 11 (73.3) | 4 (26.7) |
| Multiple (public and private) | 14 (73.7) | 5 (26.3) |
| **NCHS rural-urban category** |  |  |
| Large central metro | 71 (49.0) | 74 (51.0) |
| Large fringe metro | 38 (66.7) | 19 (33.3) |
| Medium metro | 11 (35.5) | 20 (64.5) |
| Small metro, micropolitan and non-core | 26 (66.7) | 13 (33.3) |
| **Census region** |  |  |
| Northeast | 27 (56.3) | 21 (43.8) |
| Midwest | 28 (57.1) | 21 (42.9) |
| South | 52 (53.1) | 46 (46.9) |
| West | 41 (51.9) | 38 (48.1) |
| **STI diagnosis in past 12 months** |  |  |
| No | 122 (52.1) | 112 (47.9) |
| Yes | 26 (65.0) | 14 (35.0) |
| **Condomless anal sex in past 12 months** |  |  |
| No | 55 (49.5) | 56 (50.5) |
| Yes | 93 (57.1) | 70 (42.9) |
| **Condomless vaginal sex in past 12 months** |  |  |
| No | 104 (53.9) | 89 (46.1) |
| Yes | 44 (54.3) | 37 (45.7) |
| **Number of partners** |  |  |
| One | 15 (57.7) | 11 (42.3) |
| More than one | 132 (54.3) | 111 (45.7) |
| **Marijuana use in past 12 months** |  |  |
| No | 85 (55.6) | 68 (44.4) |
| Yes | 63 (52.1) | 58 (47.9) |
| **Other non-injection illicit drug use in past 12 months** | |  |
| No | 85 (50.9) | 82 (49.1) |
| Yes | 63 (58.9) | 44 (41.1) |
| **Taking daily prescription pills** |  |  |
| No | 39 (63.9) | 22 (36.1) |
| Yes | 108 (50.9) | 104 (49.1) |
| **Injection of prescribed medication in past 12 months** |  |  |
| No | 67 (53.6) | 58 (46.4) |
| Yes, I injected myself | 44 (49.4) | 45 (50.6) |
| Yes, someone else gave me the injection | 23 (59.0) | 16 (41.0) |
| Yes, injected myself and by someone else | 13 (65.0) | 7 (35.0) |
| **Heard of LAI PrEP** |  |  |
| No | 48 (41.7) | 67 (58.3) |
| Yes | 98 (62.8) | 58 (37.2) |
| **Current PrEP prescription medication** | |  |
| Truvada | 100 (55.9) | 79 (44.1) |
| Descovy | 42 (48.8) | 44 (51.2) |
| **PrEP dose in last 30 days** |  |  |
| <15 | 53 (63.9) | 30 (36.1) |
| 16-29 | 41 (63.1) | 24 (36.9) |
| 30 | 49 (43.8) | 63 (56.3) |
| **PrEP duration** |  |  |
| Less than 2 months | 18 (51.4) | 17 (48.6) |
| 2 to 6 months | 53 (58.9) | 37 (41.1) |
| 7 to 12 months | 19 (42.2) | 26 (57.8) |
| 12 months or more | 58 (56.9) | 44 (43.1) |
