# Supplementary material for: Preferences and willingness for starting daily, on-demand, and long-acting injectable HIV pre-exposure prophylaxis among transfeminine persons in the US, 2022–2023
Source: PLoS One. 2025 Apr 1;20(4):e0320961. doi: 10.1371/journal.pone.0320961 (PMC11960950; doi:10.1371/journal.pone.0320961)
Supplement: S4 Table — (DOCX) [file pone.0320961.s004.docx]

**S4 Table.** Willingness to switch to on-demand PrEP among transfeminine persons who are current oral PrEP users, TWIST, 2022-23

|  | **Willing to switch to on-demand PrEP n (%)** | **Not willing to switch to on-demand PrEP or not sure n (%)** |
| --- | --- | --- |
| **Total** | 128 (47.1 %) | 144 (52.9 %) |
| **Age (years)** |  |  |
| 15-24 | 14 (33.3) | 28 (66.7) |
| 25-29 | 40 (45.5) | 48 (54.5) |
| 30-39 | 56 (56.0) | 44 (44.0) |
| 40+ | 18 (42.9) | 24 (57.1) |
| **Race/Ethnicity** |  |  |
| Black, non-Hispanic | 37 (77.1) | 11 (22.9) |
| Hispanic or Latino | 12 (48.0) | 13 (52.0) |
| White, non-Hispanic | 72 (40.7) | 105 (59.3) |
| Other or multipl races | 6 (28.6) | 15 (71.4) |
| **Health insurance** |  |  |
| None | 5 (50.0) | 5 (50.0) |
| Private only | 59 (39.1) | 92 (60.9) |
| Public only | 40 (54.1) | 34 (45.9) |
| Other | 7 (46.7) | 8 (53.3) |
| Multiple (public and private) | 15 (78.9) | 4 (21.1) |
| **NCHS rural-urban category** |  |  |
| Large central metro | 56 (38.9) | 88 (61.1) |
| Large fringe metro | 35 (61.4) | 22 (38.6) |
| Medium metro | 15 (48.4) | 16 (51.6) |
| Small metro, micropolitan and non-core | 20 (52.6) | 18 (47.4) |
| **Census region** |  |  |
| Northeast | 20 (41.7) | 28 (58.3) |
| Midwest | 24 (49.0) | 25 (51.0) |
| South | 55 (57.3) | 41 (42.7) |
| West | 29 (36.7) | 50 (63.3) |
| **STI diagnosis in past 12 months** |  |  |
| No | 108 (46.4) | 125 (53.6) |
| Yes | 20 (51.3) | 19 (48.7) |
| **Condomless anal sex in past 12 months** | | |
| No | 50 (45.9) | 59 (54.1) |
| Yes | 78 (47.9) | 85 (52.1) |
| **Condomless vaginal sex in past 12 months** | | |
| No | 95 (49.7) | 96 (50.3) |
| Yes | 33 (40.7) | 48 (59.3 |
| **Number of partners** |  |  |
| One | 14 (56.0) | 11 (44.0 |
| More than one | 113 (46.7) | 129 (53.3) |
| **Marijuana use in past 12 months** |  | |
| No | 81 (53.6) | 70 (46.4) |
| Yes | 47 (38.8) | 74 (61.2) |
| **Other non-injection illicit drug use in past 12 months** |  |  |
| No | 80 (48.5) | 85 (51.5) |
| Yes | 48 (44.9) | 59 (55.1) |
| **Taking daily prescription pills** |  | |
| No | 45 (76.3) | 14 (23.7) |
| Yes | 82 (38.7) | 130 (61.3) |
| **Injection of prescribed medication in past 12 months** |  |  |
| No | 68 (55.3) | 55 (44.7) |
| Yes, I injected myself | 23 (25.8) | 66 (74.2) |
| Yes, someone else gave me the injection | 27 (69.2) | 12 (30.8) |
| Yes, injected myself and by someone else | 9 (45.0) | 11 (55.0) |
| **Heard of on-demand PrEP** |  |  |
| No | 36 (35.3) | 66 (64.7) |
| Yes | 90 (53.6) | 78 (46.4) |
| **Current PrEP prescription medication** |  |  |
| Truvada | 86 (48.3) | 92 (51.7) |
| Descovy | 38 (44.7) | 47 (55.3) |
| **PrEP dose in last 30 days** |  |  |
| <15 | 62 (75.6) | 20 (24.4) |
| 16-29 | 36 (55.4) | 29 (44.6) |
| 30 | 25 (22.5) | 86 (77.5) |
| **PrEP duration** |  |  |
| Less than 2 months | 11 (31.4) | 24 (68.6) |
| 2 to 6 months | 54 (61.4) | 34 (38.6) |
| 7 to 12 months | 21 (46.7) | 24 (53.3) |
| 12 months or more | 41 (40.2) | 61 (59.8) |
